# A xenotransplantation model for reactivation of paternal *UBE3A* using human-specific antisense oligonucleotides

**DOI:** 10.1038/s41598-026-41197-9

**Published:** 2026-02-28

**Authors:** Hilde Smeenk, Bas Lendemeijer, Mirle G. Buurma, Michell A. Forgione, Denise E. Slump, Roos A. Monshouwer, Ilse Wallaard, Edwin J. Mientjes, Witte J. G. Hoogendijk, Ype Elgersma, Femke M. S. de Vrij, Steven A. Kushner

**Affiliations:** 1 Department of Psychiatry, Erasmus MC University Medical Center, Rotterdam, The Netherlands; 2 Stavros Niarchos Foundation (SNF) Center for Precision Psychiatry & Mental Health, Columbia University, New York, NY USA; 3 Department of Clinical Genetics, Erasmus MC University Medical Center, Rotterdam, The Netherlands; 4 Erasmus MC Expertise Center for Neurodevelopmental Disorders (ENCORE), Erasmus MC University Medical Center, Rotterdam, The Netherlands; 5 Department of Psychiatry, Columbia University Irving Medical Center, New York, NY USA

**Keywords:** Biological techniques, Biotechnology, Genetics, Neuroscience, Stem cells

## Abstract

For many neurodevelopmental disorders, genetic treatment discovery is complicated by non-homology between human and non-human model system genomes. Antisense oligonucleotides (ASOs) influence protein expression by sequence-specific binding of RNA, resulting in targeted degradation or alternative splicing, and thereby provide a promising avenue for clinical therapeutics. Angelman Syndrome (AS), caused by loss-of-function of maternal *UBE3A*, has emerged as a monogenic neurodevelopmental disorder for which ASO therapy might be particularly amenable due to genomic imprinting. In an AS mouse model, *Ube3a* reactivation of the paternal allele using ASOs has been previously shown to rescue disease-relevant phenotypes. However, the poor sequence homology between murine and human *UBE3A-ATS* requires a human model system for screening of ASOs with clinical therapeutic potential. Here we evaluate an ASO targeting *UBE3A-ATS* using human induced pluripotent stem cell (hiPSC)-derived AS neurons in vitro and in vivo*.* We assessed the effect of ASO-treatment on cultured AS patient hiPSC-derived neurons through protein and RNA expression analysis. To increase clinical translatability, we designed a platform to evaluate this ASO in vivo by xenotransplantation of hiPSC-derived AS neurons into the brains of neonatal mice, with intracerebroventricular administration of the *UBE3A-ATS* ASO three weeks later. One week after ASO treatment, we observed in vivo reinstatement of *UBE3A* expression in xenotransplanted human neurons. These results highlight the potential for a versatile platform to evaluate efficacy of human-specific ASOs, for which AS provides an instructive proof-of-concept for therapeutic reactivation of UBE3A.

**Supplementary Information:**

The online version contains supplementary material available at 10.1038/s41598-026-41197-9.

## Introduction

Angelman Syndrome (AS) is a severe neurodevelopmental disorder (NDD) affecting roughly 1 in 15,000 people. Symptoms include developmental delay, movement disorders, inappropriate frequent laughter, speech impairments, and severe intractable epilepsy^1^. AS is caused by loss-of-function of the maternally expressed copy of *UBE3A*, leading to the absence of functional HECT E3 ubiquitin ligase UBE3A in mature neurons^2^. Accordingly, *Ube3a* reactivation at an early age has been shown to rescue multiple behavioral phenotypes in an AS mouse model^3^. E3 ubiquitin ligases are known to tag proteins with ubiquitin, marking them for proteasomal degradation, altering localization or impacting their functioning^4^. Yet, it remains unclear mechanistically why loss of UBE3A in neurons leads to the symptoms observed in patients, as neither a defined neural circuit, disease-related molecular pathway, nor causal UBE3A ubiquitination target has been identified.

The *UBE3A* gene is located within the 15q11-q13 AS/Prader-Willi Syndrome (PWS) genomic locus. Several genes in this region exhibit parent-of-origin imprinting, with expression exclusively from the maternal or paternal allele. In most neurons of the central nervous system, *UBE3A* is exclusively expressed from the maternal allele; whereas expression is biallelic in all other cell types. On the maternal allele, CpG islands in the PWS imprinting center are methylated, thereby preventing expression of *SNURF-SNRPN* from the maternal allele (Fig. 2A). Specifically in neurons, the paternally expressed *SNURF-SNRPN* transcript is extended to form the long non-coding *UBE3A antisense* (*UBE3A-ATS*) RNA, which physically obstructs expression of paternal *UBE3A*^5–7^. Therefore, targeted degradation of the *UBE3A-ATS* could be a potential method to reactivate paternal *UBE3A* in AS neurons.

A promising approach for targeted degradation of the *UBE3A-ATS* is through antisense oligonucleotides (ASOs), short (~ 15–22 nucleotide) single-stranded nucleic acids with sequence-specific RNA binding. Depending on the sequence and chemistry, ASOs can be designed to induce targeted RNA degradation or splicing modifications^8^. ASOs are a potential therapeutic option for AS and other genetic disorders that might benefit from reducing or modifying expression of a specific RNA transcript. As groundbreaking examples, ASOs have yielded highly successful therapies for patients with spinal muscular atrophy^9^ and Duchenne muscular dystrophy^10^. For AS, the therapeutic potential of ASOs targeting the human *UBE3A-ATS* transcript has been widely appreciated. A recent Phase I clinical trial of a *UBE3A-ATS* ASO for AS showed that the ASO met safety and tolerability limits, and demonstrated modest clinical improvement in ASO-treated AS patients, suggesting that ASOs could indeed provide a promising treatment strategy for AS^11^.

Besides ASOs, small molecules could also be employed to target the *UBE3A-ATS.* Earlier studies have tested whether small molecules could be used to target *UBE3A-ATS* for *UBE3A* reactivation from the paternal allele. One of those studies used topoisomerase inhibitors, and showed that these successfully reactivated *Ube3a* from the paternal allele. However, dose-limiting cytotoxicity has thus far prevented the use of topoisomerase inhibitors for clinical applications^12^. A recent promising study identified (S)-PHA533533, a cyclin-dependent kinase 2 inhibitor to restore *UBE3A* expression through *UBE3A-ATS* downregulation in an AS mouse model and AS patient human induced pluripotent stem cell (hiPSC)-derived neurons^13^. In contrast to topoisomerase inhibition, (S)-PHA533533 appeared to have specificity for reducing *UBE3A-ATS* expression compared to other long noncoding RNAs, and is currently being considered for its clinical potential. However, given the broad potential of ASO therapy across a wide range of neurologic and neurodevelopmental disorders, we focused on reactivation of *UBE3A* as a proof-of-principle for a xenotransplantation-based platform to facilitate in vivo evaluation of molecular and cellular target efficacy.

Hybridization of an ASO with complementary RNA transcripts occurs in a sequence-specific manner through Watson-Crick base pairing. Due to species differences in the target RNA sequence, which is especially prevalent in non-coding transcripts with low evolutionary conservation, therapeutic ASOs can be challenging to screen and investigate in a non-human genomic context. This issue has been a considerable barrier to AS therapeutic discovery efforts for designing ASOs targeting human *UBE3A-ATS*^14,15^, given the almost non-existent homology to the murine *Ube3a-ATS* gene^16^. Therefore, several studies have utilized hiPSC-derived AS neurons to investigate ASOs for clinical therapeutic potential. ASO targeting of the *UBE3A-ATS* has been shown to successfully reactivate the paternal *UBE3A* allele in human iPSC-derived AS neurons^17^. Another study confirmed these findings, and performed in vivo testing in macaques, due to evolutionary divergence of the *UBE3A-ATS* gene sequence in animal models other than non-human primates^16,18^. In vivo administration remains the gold standard in clinical therapeutic development, as similar administration routes to those employed in human clinical trials can be used to assess safety and efficacy. Having non-human primates as the only available animal model greatly reduces the flexibility of experimental design, indicating a need for additional models to test human-specific genetic treatments.

Here, we leverage hiPSCs to study the effectiveness of therapeutic ASOs in an in vivo xenotransplantation model. We utilized this approach to manipulate *UBE3A* expression in human neurons derived from AS patients or healthy individuals after xenotransplantation of hiPSC-derived AS neurons into the murine brain. Treatment of brain disorders is often complicated by the blood-brain-barrier that tightly regulates the entry of compounds to the brain^19^. To overcome this, ASO treatments for disorders of the central nervous system are typically delivered directly into the cerebrospinal fluid of patients^9^. With the intention of incorporating the delivery method used in the clinic into our model, ASOs specifically targeting the human *UBE3A-ATS* transcript were delivered via intracerebroventricular (ICV) injection to restore UBE3A levels in the xenotransplanted human neurons. This model can be leveraged to validate pre-clinical ASO efficacy using human neurons in an in vivo context, moving towards precision medicine for ultra-rare genetic NDDs.

## Results

### UBE3A levels in developing hiPSC-derived neurons

**Fig. 1:**
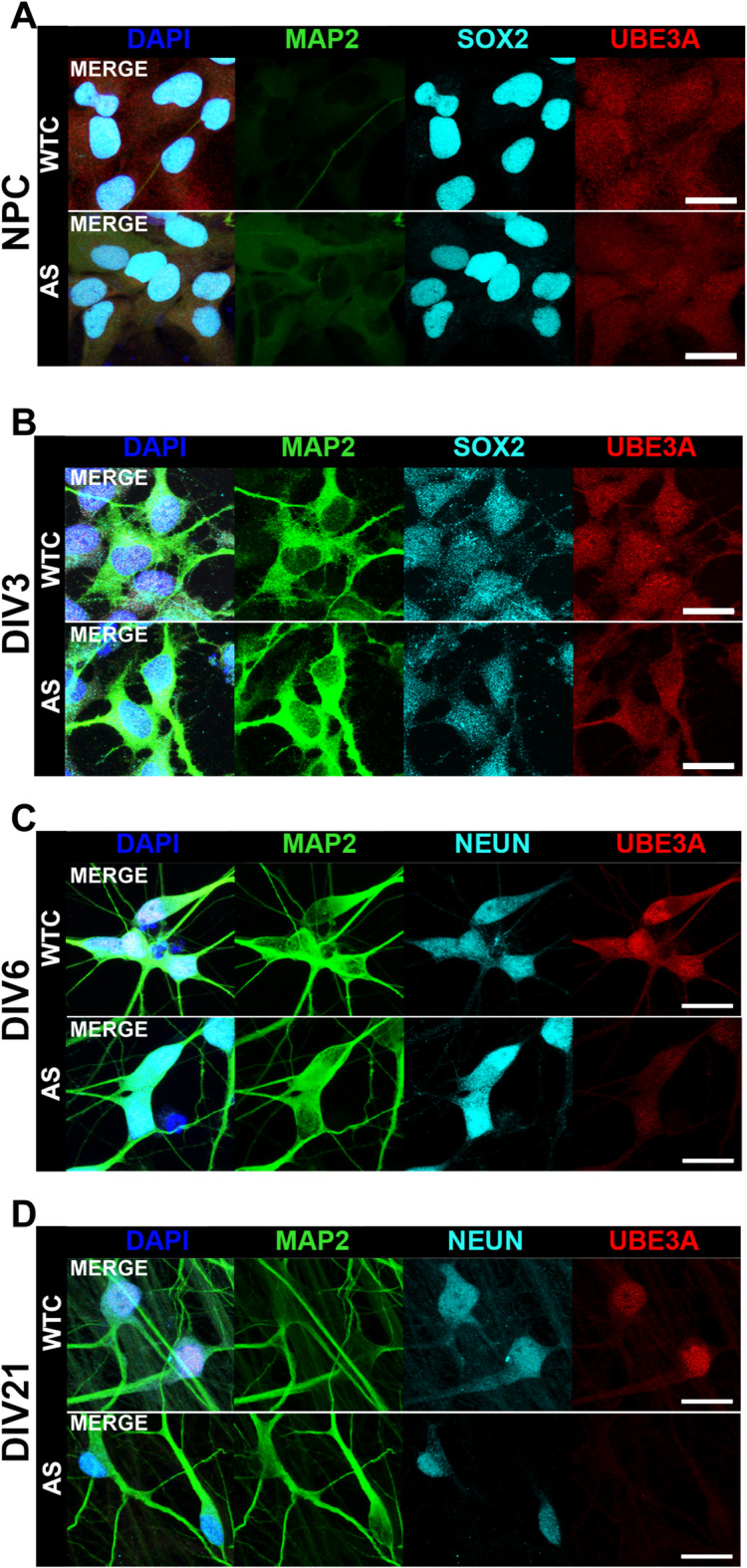
Ontogeny of UBE3A expression and subcellular localization during neuronal differentiation, (**A**) NPCs from AS Line 2 generated according to Gunhanlar et al. 2018^21^, from an AS patient and a healthy control (WTC) stain positive for the canonical neural progenitor marker SOX2 (cyan), and only show very faint staining of the neuronal marker MAP2 (green) (scale bar = 20 μm). At the progenitor stage both cell lines show UBE3A staining (red). (**B**) Neurons from AS Line 1 generated through *Ngn2* overexpression 3 days after starting differentiation. The cells express MAP2 (green), and faint signal of the neural marker NeuN (cyan) is observed, and UBE3A (red) is observed in both cell lines (scale bar = 20 μm). (**C**) After one week of differentiation, a difference is observed in UBE3A staining (red) between WTC and AS Line 1 neurons, which show clear nuclear signal of the neural marker NeuN (cyan) (scale bar = 20 μm). (**D**) Three weeks into neural differentiation, WTC neurons show clear nuclear UBE3A staining (top panel), but AS Line 1 neurons (bottom panel) no longer show UBE3A signal (scale bar = 20 μm).

Previous work using hiPSCs derived from an AS patient demonstrated that the silencing of paternal *UBE3A* expression occurs relatively late during neuronal development^20^. To verify this claim and confirm the integrity of the methylation status of the PWS imprinting center, we reprogrammed hiPSCs from two AS patient siblings (Line 1: male; Line 2: female) carrying an identical nonsense mutation in the maternal copy of *UBE3A* (W577X), along with a reference control from an unrelated healthy individual (WTC). Human iPSC lines were differentiated towards neural progenitor cells (NPCs) using a previously established embryoid-body (EB) based protocol^21^. Both WTC and AS patient-derived NPCs showed UBE3A expression in cells that were positive for SOX2, a pluripotency marker also expressed in neural stem cells (Fig. 1A). We then generated *Ngn2*^+^*/rtTA*^+^ cell lines from the hiPSC clones, to obtain forebrain excitatory neuronal cultures^22^. At DIV3, no clear difference was observed in UBE3A staining between AS patient and WTC neurons (Fig. 1B), comparable to the staining observed in NPCs. After one week of differentiation, WTC neurons showed clear UBE3A signal in the nuclei of MAP2^+^/NeuN^+^ neurons, whereas UBE3A signal was difficult to detect in the nuclei of AS-patient derived MAP2^+^/NeuN^+^ neurons (Fig. 1C). At 3 weeks of differentiation, AS patient-derived neurons (MAP2^+^/NeuN^+^) exhibited no observable expression of UBE3A (Fig. 1D), consistent with the expected *UBE3A* parent-of-origin imprinting.

### ASOs modify UBE3A expression in hiPSC-derived neurons

**Fig. 2:**
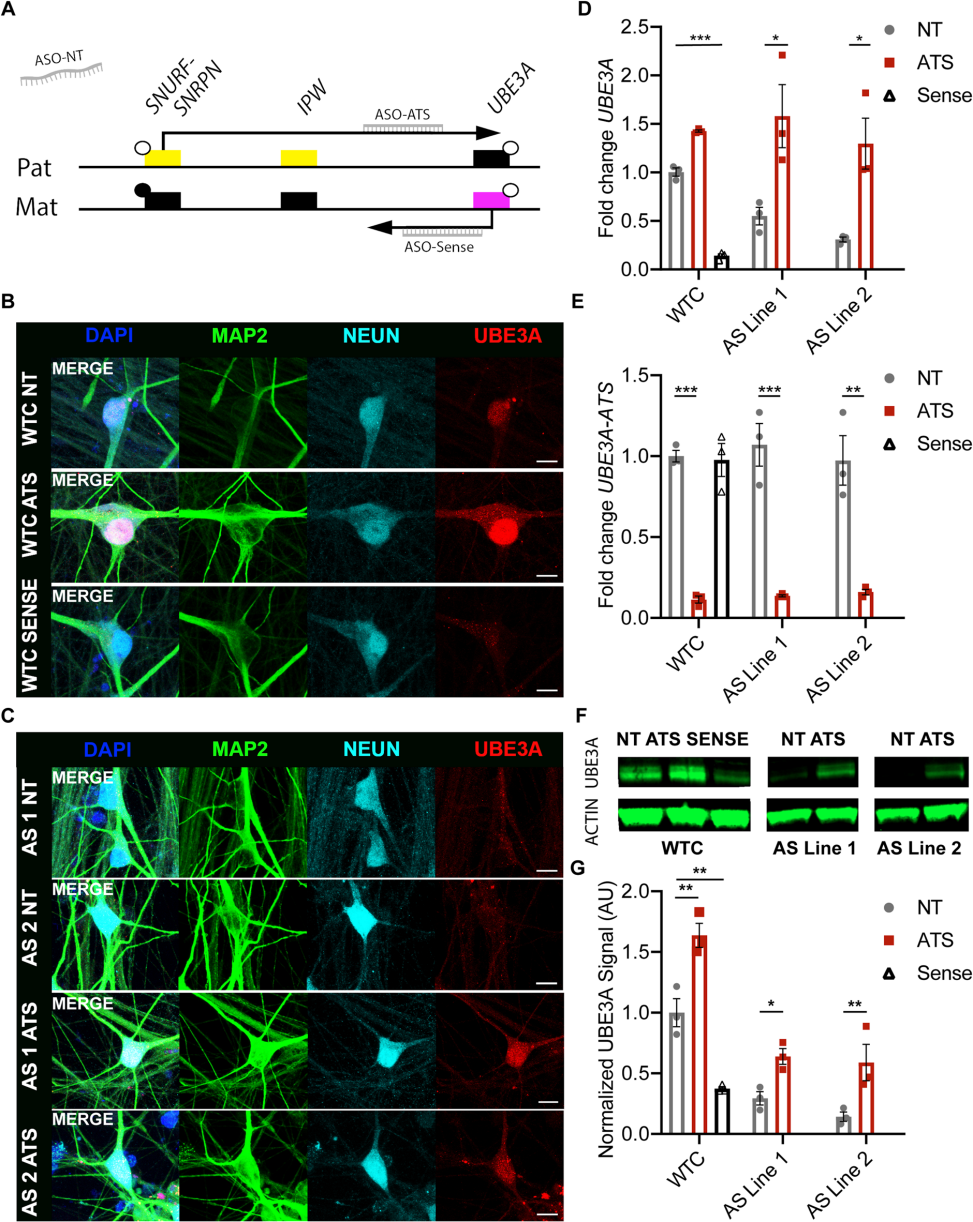
ASO treatment can be used to manipulate *UBE3A* expression in hiPSC-derived neural cultures in vitro. **(A)** Schematic representation of genomic imprinting of chromosome 15q11-q13 in neuronal cells. ASOs were designed targeting the *UBE3A-Sense* or *ATS* transcript, the non-targeting (NT) ASO has no known homologous sequence in the human genome. **(B)** WTC cultures show nuclear UBE3A (red) in mature neurons that express MAP2 (green) and NeuN (cyan) when exposed to a NT ASO (first row). Reactivation of the paternal *UBE3A* allele in WTC neurons with an *UBE3A-ATS* ASO increases UBE3A signal, surpassing baseline levels (second row). An ASO targeting the *UBE3A-Sense* transcript strongly reduces UBE3A signal from the nucleus of these cells (third row) (scale bars = 10 μm). (**C**) Mature AS neurons show no change in UBE3A levels when exposed to a NT ASO (first and second row), a *UBE3A-ATS* ASO substantially increased UBE3A levels in the nucleus (third and fourth row) (scale bars = 10 μm). (**D**) Fold change in *UBE3A* expression measured by RT-qPCR after ASO treatment. Bars indicate average ± SEM. We observe significant differences between WTC NT and WTC Sense, and *UBE3A-ATS* ASO treatment led to a significant increase in *UBE3A* expression in both AS lines, *n* = 3 replicates. (**E**) RT-qPCR for *UBE3A-ATS* transcript showing fold change after ASO treatment. Bars indicate mean ± SEM, *n* = 3 replicates. Treatment with the *UBE3A-ATS* ASO lead to a consistent decrease in *UBE3A-ATS* transcript in all cell lines, whereas treatment with the *UBE3A-Sense* ASO did not alter *UBE3A-ATS* levels. (**F**) Western blot of WTC and AS neurons at 3 weeks of differentiation, after one week of ASO treatment. Bands indicate Actin (45 kDa) and UBE3A (100 kDa). (**G**) Quantification of UBE3A protein levels from Western blot relative to Actin, bars indicate mean ± SEM. Significant differences were observed between WTC NT and both AS lines in the NT condition, as well as in all lines between NT and ATS ASO treatment. *N* = 3 independent blots with 1 biological replicate each per condition, per blot.

To confirm the in vitro modification of *UBE3A* expression, we employed an ASO targeting the *UBE3A-ATS* transcript, another targeting the *UBE3A* (sense) transcript, and a non-targeting (NT) ASO as a control (Fig. 2A). WTC and AS neurons were differentiated for 3 weeks, a timepoint at which we observed *UBE3A* imprinted expression to have occurred in all neurons (Fig. 1). Next, 2-week old cultures were treated with ASOs through supplementation in the medium for 1 week at a concentration of 5 µM. RT-qPCR of neural cultures treated with either vehicle or the NT ASO revealed no difference in *UBE3A* or *UBE3A-ATS* transcript levels (Supplementary Fig. 1B,C). UBE3A protein levels were also unchanged in cultures exposed to either vehicle or the NT ASO (Supplementary Fig. 1D,E). WTC neurons treated with the NT ASO showed the expected nuclear UBE3A signal (Fig. 2B). Treatment with the *UBE3A-Sense* ASO resulted in a loss of nuclear UBE3A expression, rendering the UBE3A expression level of treated WTC neurons similar to AS neurons (Fig. 2). WTC neurons treated with the *UBE3A-ATS* ASO exhibited an increase in UBE3A. We did not observe any obvious signs of increased cell death after treatment with ASOs in vitro.

Next, we wanted to test whether this approach could reinstate UBE3A expression in AS neurons. As expected, nuclear UBE3A was not restored in AS neurons by application of the NT ASO (Fig. 2C). In contrast, after treatment with the *UBE3A-ATS * ASO, we observed clear nuclear UBE3A labeling in AS neurons (Fig. 2C), suggesting that the *UBE3A-ATS* ASO can successfully reactivate *UBE3A* from the paternal allele in AS neurons in vitro. Quantification of nuclear UBE3A fluorescence intensity showed an increase in WTC and AS neurons treated with ATS ASO compared to NT ASO-treated cultures (fold change relative to WTC NT: WTC NT 1.00 ± 0.12; WTC ATS 1.33 ± 0.09; AS NT 0.18 ± 0.02; AS ATS 0.90 ± 0.07) (Supplementary Fig. 1A). To confirm these observations, we performed RT-qPCR for *UBE3A* and *UBE3A-ATS* transcripts on lysates of neural cultures treated with ASOs (Fig. 2D). As expected, we observed a significant increase of *UBE3A* in both AS lines after treatment with the *UBE3A-ATS* ASO compared to the AS lines treated with NT ASO (fold change relative to WTC NT: AS Line 1 NT: 0.55 ± 0.09; AS Line 1 ATS: 1.58 ± 0.32; AS Line 2 NT: 0.31 ± 0.02; AS Line 2 ATS: 1.30 ± 0.26; 2-way ANOVA: Line 1 *p* = 0.01; Line 2 *p* = 0.01). Treatment with NT ASO compared to application of vehicle to neural cultures did not affect *UBE3A* or *UBE3A-ATS* levels (Supplementary Fig. 1B,C). Although we observed a decrease in *UBE3A* between WTC and AS lines in the respective NT control conditions, this was not significant (fold change relative to WTC NT: WTC NT: 1.00 ± 0.04; AS Line 1 NT: 0.55 ± 0.09; AS Line 2 NT: 0.31 ± 0.02; 2-way ANOVA: *p* = 0.18) (Fig. 2D). We did observe significant differences among WTC neurons treated with NT versus *UBE3A-*S*ense* ASOs (fold change relative to WTC NT; WTC NT: 1.00 ± 0.04; WTC Sense: 0.14 ± 0.02; T-test: *p* < 0.01). We also performed RT-qPCR to evaluate levels of *UBE3A-ATS* in all treatment conditions (Fig. 2E). In line with expectations, we did not find differences in *UBE3A-ATS* levels between WTC and AS neurons of both lines in the NT condition (fold change relative to WTC NT: WTC NT: 1.00 ± 0.03; WTC ATS: 0.11 ± 0.02; AS Line 1 NT: 1.07 ± 0.13; AS Line 1 ATS: 0.13 ± 0.01; AS Line 2 NT: 0.97 ± 0.15; AS Line 2 ATS: 0.16 ± 0.02; 2-way ANOVA: *p* = 0.85), or in WTC neurons treated with the *UBE3A-Sense* ASO (fold change relative to WTC NT: WTC NT: 1.00 ± 0.03; WTC Sense: 0.97 ± 0.1; T-test: *p* = 0.84). In all three lines, treatment with the *UBE3A-ATS* ASO led to a significant reduction in *UBE3A-ATS* (2-way ANOVA, *p* < 0.01). To investigate how these transcriptional effects translated to protein levels, we performed Western blots on DIV21 neurons treated with ASOs during the 3rd week of differentiation (Fig. 2F and G, Supplementary Fig. 1D,E). At the protein level, we observed the expected decrease of UBE3A levels in AS neurons (AS line 1: 29.4 ± 5.4% of WTC NT, AS line 2: 14.3 ± 3.9%, 2-way ANOVA: *p* < 0.03). We also observed a significant decrease in UBE3A levels of WTC neurons treated with the *UBE3A-Sense* ASO (37.4 ± 1.7% of wildtype protein, unpaired T-test, *p* < 0.01), and a significant increase in UBE3A after treatment with the *UBE3A-ATS* ASO (163.7 ± 9.9%, 2-way ANOVA: *p* < 0.01). We found a significant increase in protein levels in the AS lines administered with *UBE3A-ATS* compared to AS neurons with the NT ASO (AS Line 1: 63.9 ± 6.4% of wildtype protein, *p* = 0.03; AS Line 2: 58.9 ± 15% of wildtype protein, *p* < 0.01; both 2-way ANOVA). Taken together, these data confirm that *UBE3A* levels can be readily manipulated using ASOs in vitro, at both the transcriptional and protein level.

### In vivo ASO treatment restores UBE3A expression in human AS neurons

**Fig. 3:**
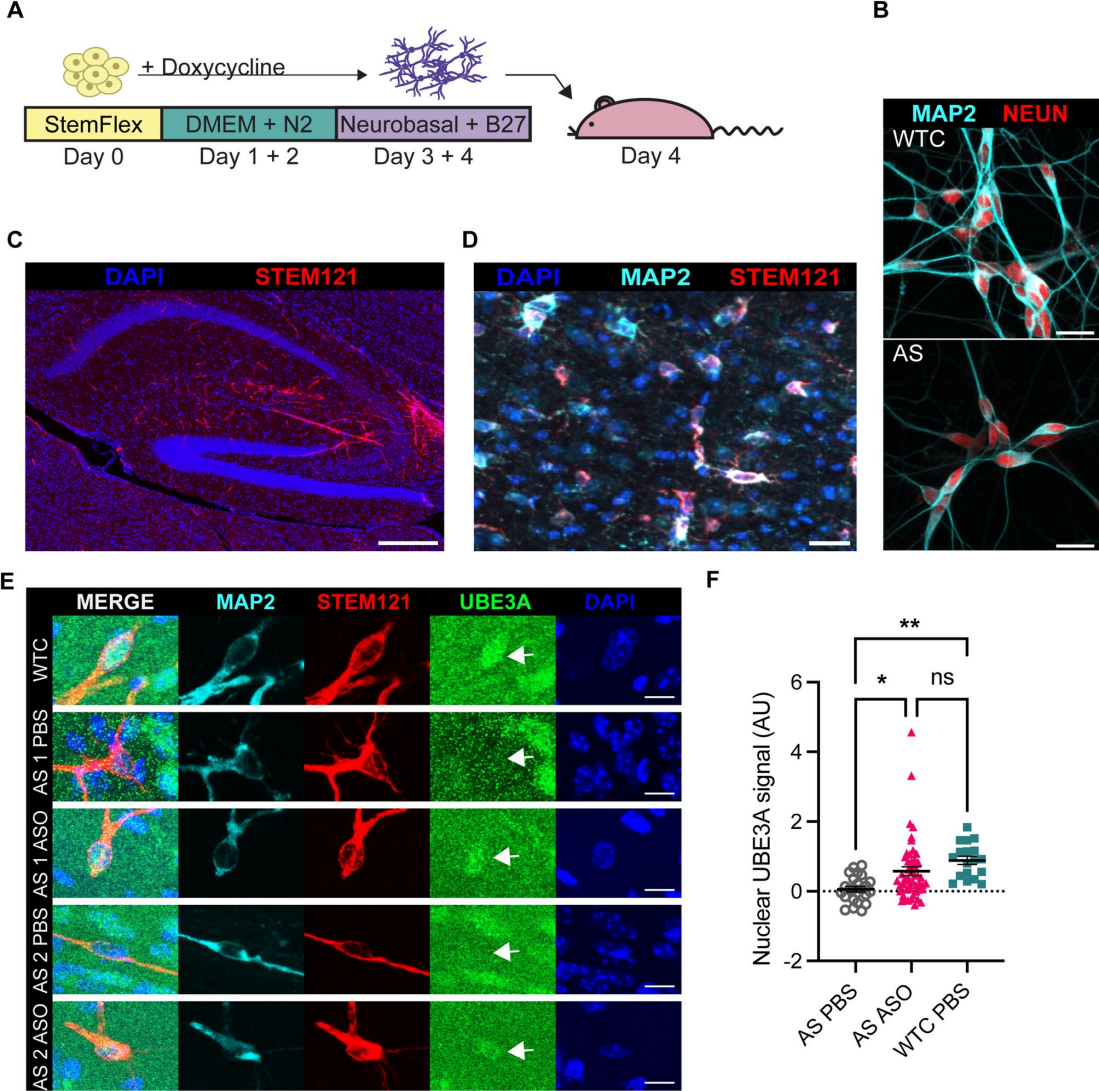
In vivo UBE3A reactivation in human AS patient-derived neurons. (**A**) A schematic overview of the xenotransplantation procedure. (**B**) WTC and AS neurons express neuronal markers MAP2 (cyan) and NeuN (red) prior to transplantation, (scale bar = 20 μm). (**C**) Representative image, showing distribution of Stem121^+^ xenotransplanted human neurons in the mouse hippocampus, scale bar = 200 μm. (**D**) Human neurons labelled with a human-specific marker (STEM121, red) and the neuronal marker MAP2 (cyan) survive at least 10 months post-transplantation (scale bar = 20 μm) (**E**) Histological analyses of human neurons after xenotransplantation. WTC (top row, MAP2^+^ (cyan) neurons show nuclear UBE3A (green) expression. In AS neurons that were xenotransplanted in mice treated with vehicle (PBS), UBE3A expression was not detected (second and fourth row). Transplanted human AS neurons in mice treated with the *UBE3A-ATS* ASO display clear nuclear UBE3A signal (third and fifth row, scale bar = 10 μm). Arrows indicate nuclei of human neurons. (**F**) Quantification of background-corrected nuclear UBE3A signal (arbitrary units; AU) in xenotransplanted human neurons in mice sacrificed one week after ASO injection, every data point indicates one human neuronal nucleus. *N =* 25 nuclei for AS PBS, 55 nuclei for AS ASO, and 15 for WTC PBS, in 6, 11, and 4 mice, respectively.

To investigate whether these human-specific ASOs would also rescue *UBE3A* expression in vivo, we designed a platform to transplant human AS patient-derived neurons in immunodeficient *Rag2*^*−/−*^ neonatal mice at p1-3 (Fig. 3A). These neurons showed expression of neuronal markers NeuN and MAP2 prior to xenotransplantation (Fig. 3B), and migrated through the mouse brain following xenotransplantation. After 4 weeks, neurons were found throughout the mouse brain, including the hippocampus (Fig. 3C), and were able to survive beyond 10 months post-transplantation (the longest timepoint examined) (Fig. 3D). We observed transplanted human neurons mostly near fiber tracts and the ventricular zones of the mouse brain, but also in the cortex. After xenotransplantation, at p21, mice received a unilateral ICV injection of either *UBE3A-ATS* ASO or vehicle. They were sacrificed a week later to investigate the effect of ASO treatment on UBE3A levels by immunohistochemistry. No differences in body weight were observed between mice treated with ASO compared to vehicle (Supplementary Fig. 2). WTC neurons in littermate controls that received vehicle injections showed the expected nuclear UBE3A signal (Fig. 3E). Furthermore, we did not observe UBE3A expression in transplanted AS neurons after ICV injection with vehicle (Fig. 3E). In contrast, UBE3A expression was restored in human transplanted AS neurons one week after treatment with the *UBE3A-ATS* ASO. The treatment effect was consistent, as nuclear UBE3A signal was consistently observed in mice treated with *UBE3A-ATS* ASO (AS PBS: 0.06 ± 0.08, AS ASO: 0.58 ± 0.13, WTC 0.88 ± 0.12; arbitrary units (AU); One-way ANOVA: *p* < 0.01), but not in those treated with vehicle (Fig. 3F, Supplementary Fig. 3A,B). UBE3A reactivation was observed in 74.5% (41 out of 55 neurons) of *UBE3A-ATS* ASO-treated AS neurons (AS Line 1: 63.6% (14 out of 22 neurons); AS Line 2: 81.8% (27 out of 33 neurons); *n* = 11 mice treated with the *UBE3A-ATS* ASO, across 3 different litters), demonstrating that xenotransplanted human neurons are reliably targeted by intracerebroventricular ASO administration.

## Discussion

*UBE3A* levels in the brain are tightly regulated during neuronal development and maturation, for which insufficient expression of UBE3A is a major pathophysiological mechanism underlying AS^23^. The mechanisms regulating gene expression and parent-of-origin imprinting within the 15q11.2 AS/PWS genomic locus are highly conserved between species^24^; however, the human *UBE3A-ATS* sequence shows very limited conservation with most non-human species including rodents. Therefore, ASOs designed to reactivate the paternal *UBE3A* allele will almost invariably require a human genetic background to investigate. We set out to create a model in which therapeutic ASOs for human NDDs can be tested in vivo towards future implementation in the clinic. To achieve this, human neurons were transplanted into the brain of neonatal immunodeficient mice followed by intracerebroventricular administration of ASOs in recipient host mice. As a proof-of-concept, we restored UBE3A levels in xenotransplanted human AS neurons into the murine brain through intracerebroventricular delivery of an ASO targeting *UBE3A-ATS.*

In vitro, we observed a significant increase in *UBE3A* transcript levels after treatment with the *UBE3A-ATS* ASO, restoring UBE3A protein levels to approximately 60% of wildtype. We observed restoration of nuclear UBE3A in this study, highlighting the potential therapeutic effects, as loss of nuclear UBE3A is sufficient to cause AS symptoms in mice^25^. Notably, *UBE3A* was still detectable in AS neurons treated with the NT ASO, which is similarly observed in cultures of untreated AS neurons owing to small populations of immature neural lineage cells that retain biallelic *UBE3A* expression. In vivo, we did not observe UBE3A expression among xenotransplanted AS neurons, suggesting a greater level of neuronal maturity and paternal allele silencing. In an AS mouse model, similar levels of restoration after p21 injection of a murine-optimized *Ube3a-ATS* ASO were achieved in the cortex, hippocampus, and cerebellum (~ 60% of wildtype UBE3A) – with the exception of the striatum, in which full restoration was achieved^14^. This proved sufficient to rescue an audiogenic seizure phenotype in the mice, suggesting that restoration of UBE3A levels equal to wildtype levels may not be necessary for therapeutic benefit in AS.

Interestingly, cellular UBE3A levels may not correspond linearly to *UBE3A* gene dosage. UBE3A is known to auto-ubiquitinate, thereby controlling its own degradation^26^. In a mouse model of *Ube3a* gene dosage, increased *Ube3a* copy number resulted in a sublinear increase in *Ube3a* and translated protein, suggesting that cellular UBE3A levels may not only be regulated through auto-ubiquitination, but may also be regulated through transcriptional mechanisms. In 15q11-q13 duplication syndrome (Dup15q), an NDD caused by duplication of the entire 15q11-13 region around *UBE3A*, postmortem brains showed lower UBE3A levels than expected based on copy number^27^. The Dup15q phenotype also emphasizes the clinical risk of UBE3A overexpression, as increased UBE3A levels are one of the major pathogenic mechanisms in Dup15q^28^.

Numerous NDDs have been established as resulting from a well-defined genetic etiology that causes dysfunction of a single protein. For many of these disorders, ASOs offer a promising therapeutic modality. The experimental approach presented here provides a platform to test therapeutic ASOs before translation to the clinic. Effectiveness and toxicity can be evaluated on human cells while also incorporating delivery method and potential off-target effects in an in vivo context. However, toxicity mechanisms may also be non-homologous between species. We have not performed extensive toxicity and murine off-target experiments, and this requires further investigation. For future novel therapeutic ASO development, our model could provide additional understanding of the clinical potential of novel therapeutic ASOs and related toxicity mechanisms that do not rely on direct sequence conversion. A recent study used a similar approach to test ASOs on xenografted cortical organoids from Timothy Syndrome patient-derived hiPSCs^29^. Similar to our findings, restoration of expression of the therapeutic target was observed in xenotransplanted cells after intracerebroventricular ASO administration to the host rats, further validating the potential of such a model. Our approach leveraged xenotransplantation of a more homogeneous lineage-restricted population of hiPSC-derived neurons, which may be advantageous for screening therapeutics expected to disproportionately impact defined cell types. Accordingly, the nature of the particular disorder and corresponding molecular target under investigation might favor one or the other approach.

The first results of a clinical trial for Angelman Syndrome utilizing a novel ASO (Rugonersen, RO7248824) against *UBE3A-ATS* appear promising, demonstrating an acceptable safety and tolerability profile, dose-dependent partial normalization of the characteristic AS-associated electroencephalogram abnormality, and modest evidence of clinical improvement^11^. More generally, advances in sequencing techniques have led to increasingly frequent clinical diagnosis of pathogenic variants among individuals with NDDs^30^, many of which could potentially be treated using ASOs. However, since the vast majority of the variants are ultra-rare, traditional clinical trial designs are problematic as there may not be enough patients for a traditional randomized placebo-controlled clinical trial. In vivo xenotransplantation approaches could help facilitate efforts to develop N-of-1 pipelines for ASO-based ultra-rare NDD therapeutic development^31^. In light of the rapid expansion of whole genome and exome-based newborn screening^32^, it is likely that increasing numbers of infants and children will have early molecular diagnoses of NDDs, for which therapeutic efficacy may be dependent upon early intervention. A challenge for the field will be to establish well validated and robust clinical therapeutic pipelines for assessing safety and efficacy in the context of N-of-1 conditions for ultra-rare variants underlying NDDs.

## Methods

### HiPSC differentiation towards neural progenitor cells

A healthy control hiPSC-line (WTC-11, Coriell #GM25256, male, age 30) and hiPSC lines derived from skin biopsies of two siblings with AS (*UBE3A*^*+/W577X*^; Line 1, #EMC28.4: male, age 40; Line 2. #EMC31.1: female, age 39) were used in this study. Mycoplasma testing was performed monthly, and genomic integrity of hiPSCs was screened using genome-wide screening arrays every 10 passages. hiPSCs were differentiated to NPCs as previously described^21^ with minor modifications. HiPSC colonies were cultured in hES medium (Table 1) on a layer of mouse embryonic fibroblasts. Undifferentiated hiPSC colonies were dissociated from the feeder layer of a 60–70% confluent 6-well plate with Collagenase IV (ThermoFisher, 17104019). Afterwards, embryoid body formation was started by transfer of colonies to a 10 cm dish containing hES medium without fibroblast growth factor, which was kept on a shaker (+/- 50 RPM) for 2 days. On DIV2, the medium was changed to neural induction medium (Table 1) and refreshed every other day. After a week, the embryoid bodies were plated on laminin coated dishes (20 µg/ml (Sigma, L2020)). Then, on DIV14 the medium was switched to NPC medium (Table 1). Cells were passaged 1:4 every week with Collagenase IV and a cell lifter. After passage 3, fluorescence-activated cell sorting (FACS) was performed to purify NPC cultures^33^. NPCs were dissociated using accutase (Sigma, A6964) to obtain a single cell suspension. CD184^+^/CD44^−^/CD271^−^/CD24^+^ cells were isolated through the use of a FACSaria III (BD Biosciences), after which they could be expanded and cryoperserved. All cells were cultured in a humidified incubator at 37 °C with 5% CO_2_.

**Table 1:** Overview of media and reagents used during the differentiation protocol.

Name	Reagents	Manufacturer, catalogue number
hES medium	Advanced DMEM/F12	ThermoFisher Scientific, 1634010
	20% Knockout Serum Replacement	ThermoFisher Scientific, 10828028
	1% MEM-NEAA	ThermoFisher Scientific, 11140035
	7 nl/ml β-mercaptoethanol	Sigma-Aldrich, M7522
	1% L-Glutamine	ThermoFisher Scientific, 25030024
	1% Penicillin-Streptomycin	ThermoFisher Scientific, 15140122
	10 ng/ml basic Fibroblast Growth Factor	Merck, GF003AF
StemFlex medium	StemFlex medium	ThermoFisher Scientific, A3349401
	1% Penicillin-Streptomycin	ThermoFisher Scientific, 15140122
NGN2 DIV 1 Medium	Advanced DMEM/F12	ThermoFisher Scientific, 1634010
	1% N-2 Supplement	ThermoFisher Scientific, 17502048
	1% MEM-NEAA	ThermoFisher Scientific, 11140035
	1% Penicillin-Streptomycin	ThermoFisher Scientific, 15140122
	200 ng/ml Laminin	Sigma-Aldrich, L2020
	4 µg/ml Doxycycline	Sigma-Aldrich, D9891
	10 ng/ml NT-3	Stemcell Technologies, 78074-1
	10 ng/ml BDNF	ProSpec, CYT-207
NGN2 DIV 3 Medium	Neurobasal medium	ThermoFisher Scientific, 21103049
	2% B-27 minus RA Supplement	ThermoFisher Scientific, 12587010
	1% GlutaMAX Supplement	ThermoFisher Scientific, 35050061
	1% Penicillin-Streptomycin	ThermoFisher Scientific, 15140122
	4 µg/ml Doxycycline	Sigma-Aldrich, D9891
	10 ng/ml NT-3	Stemcell Technologies, 78074-1
	10 ng/ml BDNF	ProSpec, CYT-207
Neural Induction Medium	Advanced DMEM/F12	ThermoFisher Scientific, 1634010
	1% N-2 Supplement	ThermoFisher Scientific, 17502048
	2 µg/ml Heparin	Sigma-Aldrich, H3149
	1% Penicillin-Streptomycin	ThermoFisher Scientific, 15140122
NPC Medium	Advanced DMEM/F12	ThermoFisher Scientific, 1634010
	1% N-2 Supplement	ThermoFisher Scientific, 17502048
	2% B-27 minus RA Supplement	ThermoFisher Scientific, 12587010
	1 µg/ml Laminin	Sigma-Aldrich, L2020
	1% Penicillin-Streptomycin	ThermoFisher Scientific, 15140122
	20 ng/ml basic Fibroblast Growth Factor	Merck, GF003AF
Neural differentiation medium	Neurobasal medium	ThermoFisher Scientific, 21103049
	1% N-2 Supplement	ThermoFisher Scientific, 17502048
	2% B-27 minus RA Supplement	ThermoFisher Scientific, 12587010
	1% MEM NEAA	ThermoFisher Scienfitic, 11140035
	2 µg/ml Laminin	Sigma-Aldrich, L2020
	1% Penicillin-Streptomycin	ThermoFisher Scientific, 15140122
	20 ng/ml BDNF	ProSpec, CYT-207
	20 ng/ml GDNF	ProSpec, CYT-305
	1 µM db-cAMP	Sigma-Aldrich, D0627
	200 µM ascorbic acid	Sigma-Aldrich, A5960

### hiPSC differentiation towards neurons

Differentiation of hiPSC to neural cultures was performed as described before, with minor modifications^22^. In brief, hiPSCs were cultured in StemFlex medium (Thermofisher), on Matrigel^®^-coated plates. Stably integrated *Ngn2*^*+*^*/rtTA*^*+*^ hiPSC lines were generated using lentiviral vectors (pLVX-EF1α-(Tet-On-Advanced)-IRES-G418(R), pLVX-(TRE-thight)-(MOUSE)Ngn2-PGK-Puromycin(R)), after which antibiotic selection with G418 and puromycin was performed to retain colonies that stably expressed the vectors. For differentiation to neurons, hiPSCs from 80 to 90% confluent wells were dissociated using Accutase™ (Sigma, A6964), and plated in StemFlex medium supplemented with 4 µg/ml doxycycline (Sigma, D9891) and RevitaCell™ (Thermofisher, A2644501), on wells pre-coated with Matrigel. The following day, medium was switched to DIV1 media (Table 1). WTC astrocytes, differentiated from NPCs according to a previously published protocol^34^, were added on DIV2, in a 1:1 ratio. On DIV3, cell culture medium was changed to NGN2 DIV3 media (Table 1), with supplementation of 2 µM Cytarabine (Sigma-Aldrich, C1768). From DIV5 onwards, half of the medium was replaced every other day, and from DIV14 onwards, doxycycline supplementation was stopped.

### In vitro ASO treatment of hiPSC-derived neurons

ASOs used in this study were kindly provided by Roche. ASOs were all LNA-DNA gapmers with a phosphorothioate backbone. Neural cultures were treated with ASOs by bath application between weeks 2 and 3 at a concentration of 5 µM in neural differentiation medium. *UBE3A-Sense* ASO (RTR17169, 5’-TTTACACCTACTTCTTAACA-3’) targeting the *UBE3A* transcript was used to prevent translation of UBE3A protein, *UBE3A-ATS* ASO (RTR16964, 5’-CTTTCCATTTATTTCCATTT-3’) was used to induce breakdown of the non-coding ATS transcript from the paternal allele and reactivate paternal *UBE3A* expression in neurons. The non-targeting (NT) ASO (RTR22946, 5’-CCAAATCTTATAATAACTAC-3’) without sequence homology to any human transcript was used to control for potential nucleic acid-induced toxicity.

### Cell culture immunocytochemistry

Cells were fixed in 4% formaldehyde (FA) in PBS (Merck, 1040032500), and labeled using immunocytochemistry. Primary antibodies were incubated overnight at 4 °C, secondary antibodies for 2 h at room temperature. Antibody incubation was performed in staining buffer [0.05 M Tris, 0.9% NaCl, 0.25% gelatin, and 0.5% Triton X-100 (Sigma, T8787) in PBS (pH 7.4)]. A list of primary antibodies and their dilutions can be found in Table 2. Secondary antibodies conjugated to Alexa-488, Alexa-647 or Cy3 were used at a dilution of 1:400 (Jackson ImmunoResearch), and DAPI (ThermoFisher Scientific, D1306) was added to the secondary staining mix to visualize nuclei. Sample mounting was performed in Mowiol 4–88 (Sigma-Aldrich, 81381). Samples were imaged using a Zeiss LSM 800 confocal microscope (Oberkochen, Germany).

**Table 2:** Overview of primary antibodies used.

Antibody	Dilution	Manufacturer, catalogue number
Actin	1:5000	Millipore, MAB1501R
CD15 V450	1:100	BD Bioscience, 561584
CD24 PE-Cy7	1:250	BD Bioscience, 561646
CD44 FITC	1:100	BD Bioscience, 560977
CD184 APC	1:250	BD Bioscience, 560936
CD271 PE	1:500	BD Bioscience, 560927
GFAP	1:200	Millipore, AB5804
Human Nuclei	1:500	Millipore, MAB1281
MAP2	1:200	Synaptic Systems, 188004
NeuN	1:200	Millipore, ABN78
SOX2	1:200	Millipore, AB5603
STEM121	1:500	Takara Bio, Y40410
UBE3A (ICC)	1:200	Sigma, SAB1404508
UBE3A (WB)	1:1000	Sigma, E8655
UBE3A (IHC)	1:250	Bethyl, BET A300-352A-T

### Western blotting

ASO treatment was performed as mentioned above, from DIV14 to DIV21. Control samples were treated with PBS. On DIV21, samples were collected in PBS with 1:100 protease inhibitor cocktail (Sigma-Aldrich, P8340) and centrifuged for 3 min at 1000G, at 4 °C. Samples were stored at -80 °C until analysis. Prior to Western blotting, samples were reconstituted in 1x Laemmli buffer (10% Tris HCl pH 6.8, 4% SDS in dH_2_O) and protein levels were determined using a Pierce™ BCA Protein Assay Kit (Thermofisher, 23225). For the blotting, 20 µl samples containing 25 µg protein in 1x XT sample buffer (Bio-Rad, 1610791) were prepared and heated to 96 °C for 5 min. These samples were loaded onto 4–12% Criterion™ Bis-Tris Precast gels (Bio-Rad, 3450124), in a Criterion™ Vertical Electrophoresis Cell (Bio-Rad, 1656001) in 1x XT MOPS running buffer (Bio-Rad, 1610788), using the Pageruler Plus Prestained protein ladder (Thermofisher, 26619) as a size marker. Electrophoresis was started at 100 V for 15 min, after which the voltage was increased to 125 V for another 2 h. For protein transfer, the gel was placed in a Trans-Blot Turbo Midi 0.2 μm Nitrocellulose Transfer Pack (Bio-Rad, 1704159), and transferred in a Trans-Blot Turbo Transfer system (Bio-Rad 1704150), according to the manufacturer’s instructions. Next, the membrane was blocked in 4% milk in TBS-T for an hour at room temperature, after which primary antibody staining (mouse anti-actin, mouse anti-UBE3A; Table 2) was performed overnight at 4 °C, in 2% milk in TBS-T. The following day, the membrane was washed twice with TBS-T. Secondary staining (IRDye^®^ 680LT Donkey anti-Mouse IgG, 1:15000, LI-COR Biosciences, 926-68022) was performed for 2 h at room temperature, followed by washing with TBS-T three times, and then washing with TBS twice. Blots were imaged on an Odyssey CLx (LI-COR Biosciences, 9140), and image analysis was performed using Image Studio Lite (LI-COR Biosciences). UBE3A levels were quantified relative to the actin bands in the same lane, as a loading control.

### RT-qPCR

ASO treatment was performed as described above. For RNA isolation, cultures were manually detached from culture plates using a cell lifter and centrifuged for 3 min at 1000G, at 4 °C. RNA isolation was then performed using a Qiagen RNeasy Mini kit (Qiagen, 74104). 300 ng of RNA was used for cDNA synthesis, which was performed using an iScript cDNA synthesis kit (Bio-Rad, 1708891) according to manufacturer’s instructions. For qPCR, 40 ng of cDNA and 0.2 µM primer was added per reaction mix. Primers (Table 3) were diluted in SYBR Green Universal Master Mix (Thermofisher, 4309155), and samples were run on a CFX96 Touch Real-Time PCR Detection System (Bio-Rad, 1845096) on a C1000 Touch Thermal Cycler (Bio-Rad, 1841100). Per biological replicate, 3 technical replicates were run. For data analysis, Cq values were normalized to *GAPDH*, and fold changes of expression relative to WTC NT samples were calculated.

**Table 3:** Primers used in this study.

Target	Sequence
GAPDH forward	TCAAGAAGGTGGTGAAGCAGG
GAPDH reverse	ACCAGGAAATGAGCTTGACAAA
UBE3A forward	AGATGATGCACTTGTCCGGC
UBE3A reverse	TCTGCAGGATTTTCCATAGCG
UBE3A-ATS forward^38^	GCACTGAAAATGTGGCATCCAGTC
UBE3A-ATS reverse^38^	GGTGTGTCAGCTGTGCTGGTGTCA

### Neuron xenotransplantation in neonatal immunodeficient mice

Before xenotransplantation, *Ngn2*^*+*^*/rtTA*^*+*^ hiPSCs were differentiated to neurons in 4–5 days as mentioned above, without addition of astrocytes. HiPSC-derived neurons were xenotransplanted into the brains of immunodeficient neonatal (P1-3) *Rag2*^*−/−*^ BALB/c (gift from Prof. Gerard Wagemaker, Erasmus MC^35^ x *Rag2*^*−/−*^ CB6F1/J (The Jackson Laboratory, 008449) F1 hybrid mice. Xenotransplantation was performed as described before, with minor modifications^34^. Pups underwent cryoanesthesia and 5–10 × 10^4^ cells were resuspended in PBS with 1 mg/ml Fast Green FCF (Merck, F7252) and cell injections were delivered in a 1 µl drop via a 1 mm-diameter pulled glass pipette at five different regions – in the posterior and anterior anlagen of the corpus callosum bilaterally and in the cerebellar peduncle dorsally. Afterwards, pups recovered on a heating mat and were placed back in the nest with their mother upon awakening. Mice were sacrificed at 4 weeks of age by transcardiac perfusion with PBS followed by 4% PFA. Brains were isolated and incubated in 4% PFA for 2 h at room temperature, then transferred to a 10% sucrose/phosphate buffer (PB 0.1 M, pH 7.3) and stored overnight at 4°C. Brain embedding was performed in 12% gelatin/10% sucrose blocks, block fixation was performed for 2 h at room temperature in a 10% PFA/30% sucrose solution. Embedded brains were incubated in 30% sucrose/phosphate buffer (PB 0.1 M, pH 7.3) at least overnight at 4°C. Next, the brains were sectioned into 40 μm slices on a freezing microtome (Leica, Wetzlar, Germany; SM 2000 R). Sections were then pre-incubated in blocking buffer (0.5% Triton X-100 (Sigma, T8787) and 10% normal horse serum (NHS; ThermoFisher, 16050122) in PBS) for 1 h at room temperature. Primary antibody incubation was performed for 48 h at 4°C (Table 2). Secondary antibodies conjugated to Alexa-488, Alexa-647 or Cy3 were used at a dilution of 1:400 (Jackson ImmunoResearch), nuclei were visualised using DAPI (ThermoFisher Scientific, D1306) and secondary antibodies were incubated for 2 h at room temperature. Both primary and secondary antibodies were applied in staining buffer (2% NHS and 0,5% Triton X-100 in PBS). Samples were finally mounted using Mowiol 4–88 (Sigma-Aldrich, 81381) and imaged on a Zeiss LSM 800 confocal microscope (Oberkochen, Germany).

### ASO treatment of xenotransplanted mice

Treatment of xenotransplanted mice was performed using an ICV injection^36^ of 95 µg ATS ASO at P21, and littermate controls received in ICV injection with PBS. Briefly,  *Rag2*^−/−^ mice were initially anaesthetized using 5% isoflurane and placed in a stereotaxic frame (David Kopf Instruments), sedation was maintained using 1.5% isoflurane during the entire procedure. After exposing the skull, a glass needle (0.5–0.7 μm tip) was placed 0.5 mm posterior and 1.0 mm lateral to the bregma, and lowered to a depth of 1.5 mm from the meninges. A 3 µl ASO (28.3 µg/µl) or PBS injection was delivered at a rate 0.5 µl/min using a syringe pump (CMA microdialysis, CMA400). Following the injection, the needle was left in place for an additional 5 min to ensure diffusion of the compound. After slow retraction of the needle, the skin incision was sutured and the animals were left to recover under a heating lamp.

### Quantification of UBE3A fluorescent signal

Images were processed using Fiji^37^. Regions of interest were defined around the nuclei of human neurons in the DAPI channel, and integrated density measurements were taken of UBE3A antibody signal. For in vitro analysis, regions of interest were defined around NeuN^+^ nuclei. Background was subtracted from the UBE3A signal by drawing an identical region of interest nearby where no cells were present, and outlier analysis was performed using Grubb’s test.

### Statistical analyses

Statistical analyses were performed using GraphPad PRISM. For statistical analyses of qPCR and Western blot, 2-way ANOVA with Šidák’s post-hoc test was performed. For comparison of NT vs. Sense conditions, an unpaired t-test was performed. UBE3A intensity measurements were analyzed using one-way ANOVA with Tukey’s post-hoc test. For comparison of murine weight progression, a simple linear regression was performed. Results were considered statistically significant when *p* < 0.05.

## Supplementary Information

Below is the link to the electronic supplementary material.

Supplementary Material 1

## Data Availability

Data generated and analyzed in this study may be requested from the corresponding author, upon reasonable request.
