# Supplementary material for: A xenotransplantation model for reactivation of paternal UBE3A using human-specific antisense oligonucleotides
Source: Sci Rep. 2026 Feb 28;16:11443. doi: 10.1038/s41598-026-41197-9 (PMC13056972; doi:10.1038/s41598-026-41197-9)
Supplement: Supplementary file 1 — Supplementary Material 1 [file 41598_2026_41197_MOESM1_ESM.docx]

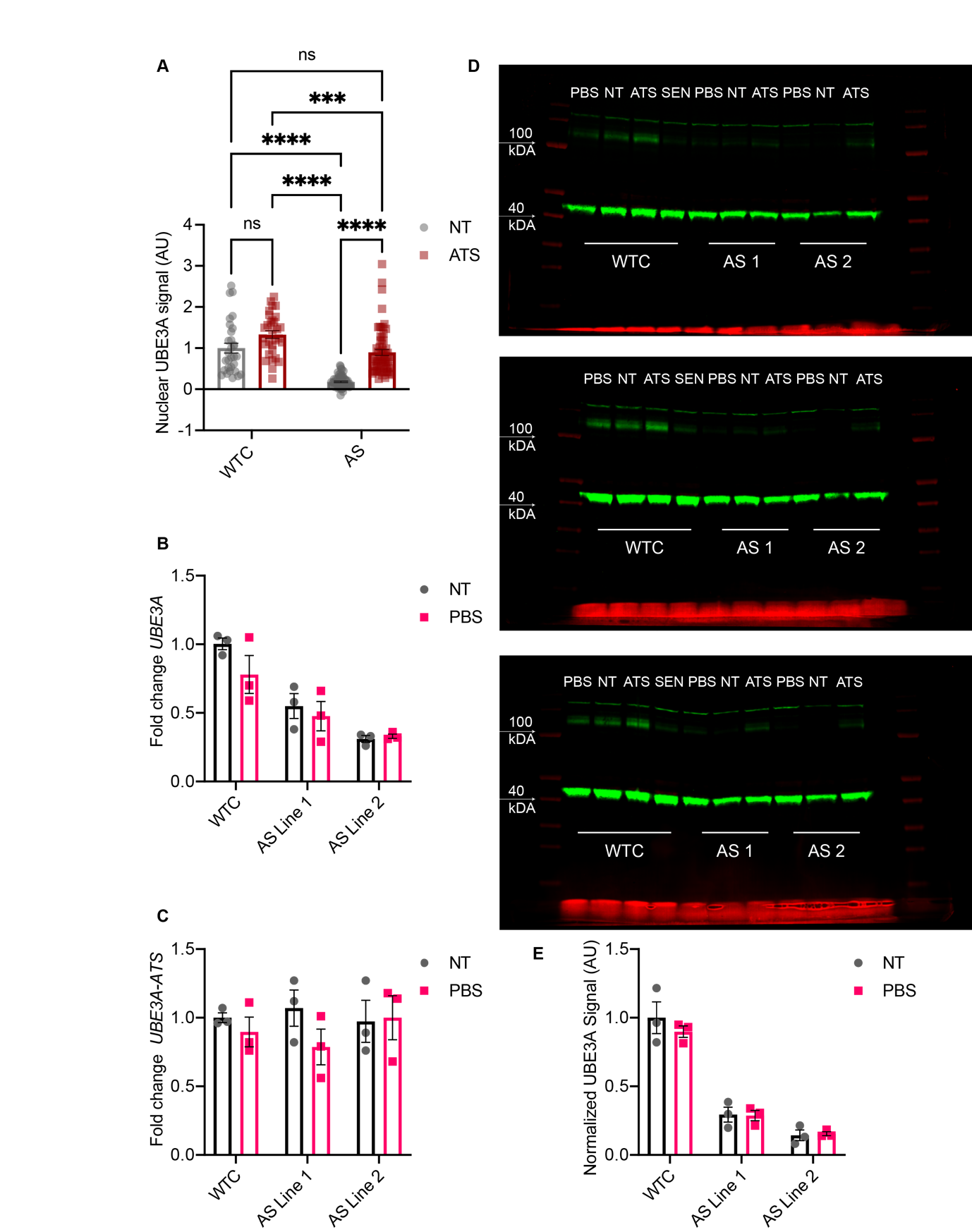


**Supplementary Figure 1**: Comparison of neurons treated with ASOs and vehicle (PBS). (**A**) Quantification of normalized nuclear UBE3A intensity relative to WTC neurons treated with NT ASO, on neural cultures treated with NT or ATS ASO, related to figures 2B and 2C. Fold change relative to WTC NT: WTC NT 1.00 ± 0.12; WTC ATS 1.33 ± 0.09; AS Line 1 NT 0.17 ± 0.03; AS Line 1 ATS 0.62 ± 0.04; AS Line 2 NT 0.19 ± 0.03; AS Line 2 ATS 1.17 ± 0.12; 2-way ANOVA, p < 0.01, *n =* 30 nuclei per condition. (**B**) RT-qPCR analysis of *UBE3A* in neural cultures versus PBS or NT ASO treatment ± SEM (2-way ANOVA with Šidák’s multiple comparisons test: treatment effect p = 0.23, *n = 3* replicates) (**C**) Fold change in *UBE3A-ATS* RNA relative to WTC treated with a NT ASO (2-way ANOVA: p = 0.26, *n* = 3 replicates) (**D**) Western blots of WTC and AS neurons treated with either PBS or NT ASO. Top bands represent UBE3A (100 kDa), bottom bands Actin (45 kDa). A nonspecific band can be observed above the UBE3A band in all conditions. *N* = 3 independent blots with one biological replicate per blot. (**E**) UBE3A protein levels measured with Western Blot, normalized to WTC NT samples, relative to Actin. No significant difference is observed between samples treated with NT ASO or PBS (2-way ANOVA: p = 0.4, *n* = 3 replicates).


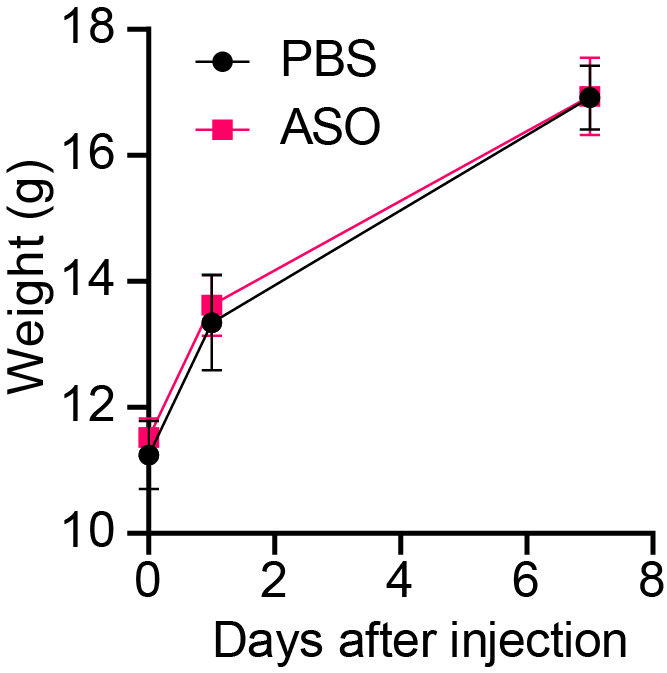


**Supplementary Figure 2**: No differences were observed in weight progression of mice treated with either *UBE3A-ATS* ASO or vehicle (PBS), (pooled slope = 0.71, p = 0.8)

**
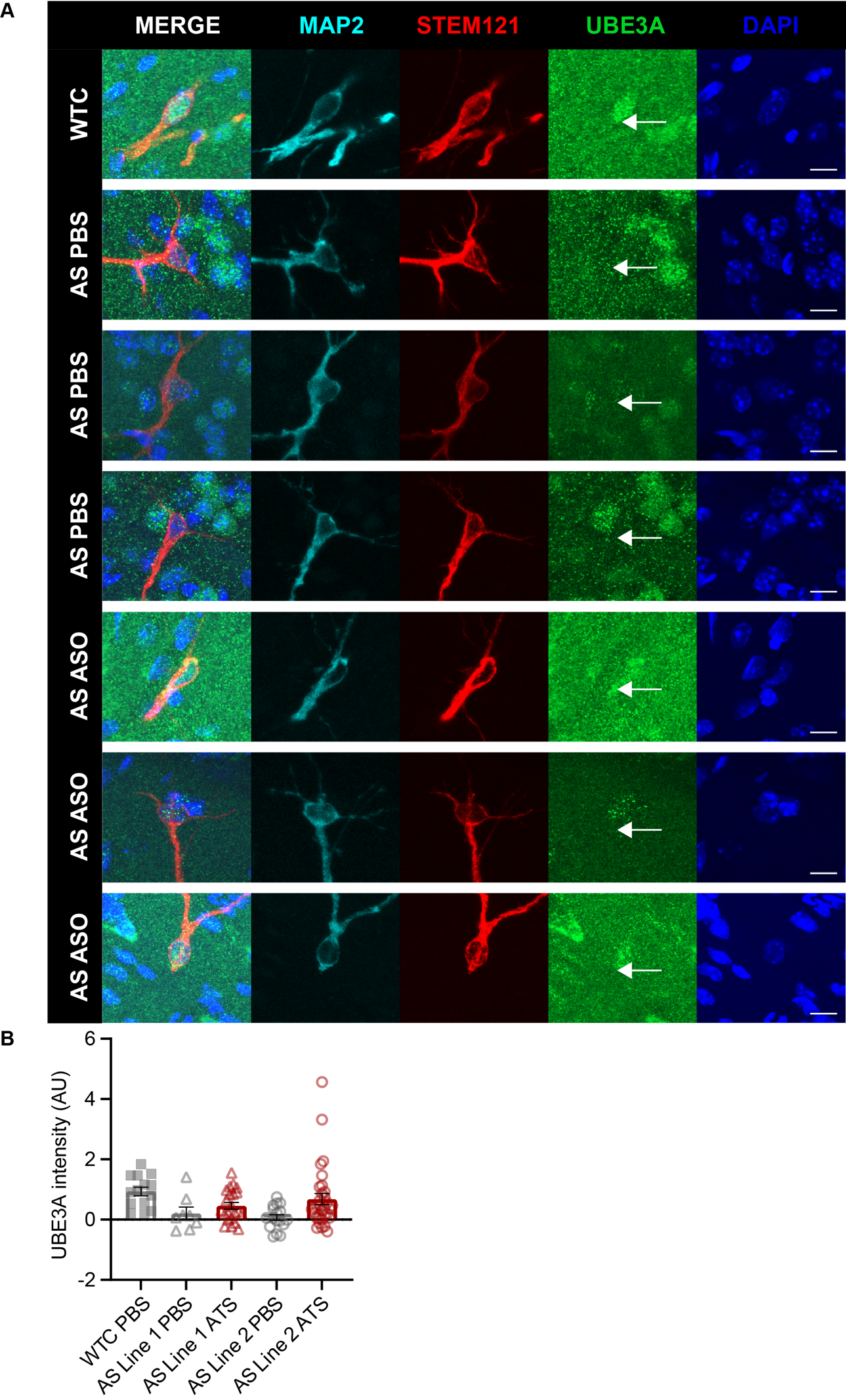
Supplementary Figure 3: (A)** Example images of transplanted human neurons in different mice from the same litter. Representative images obtained from individual mice, with neuronal marker MAP2 in cyan, human-specific marker Stem121 in red, UBE3A in green and DAPI in blue. Scale bar = 10 µm. (**B**) UBE3A nuclear fluorescence intensity in xenotransplanted neurons of either the WTC line or the two AS lines, in mice treated with vehicle (PBS) or the *UBE3A-ATS* ASO (WTC PBS 0.88 ± 0.12; AS Line 1 PBS 0.21 ± 0.21; AS Line 1 ATS 0.53 ± 0.11; AS Line 2 PBS -0.33 ± 0.15; AS Line 2 ATS 0.86 ± 0.26; arbitrary units (AU)).
